# DME-DeepLabV3+: a lightweight model for diabetic macular edema extraction based on DeepLabV3+ architecture

**DOI:** 10.3389/fmed.2023.1150295

**Published:** 2023-09-08

**Authors:** Yun Bai, Jing Li, Lianjun Shi, Qin Jiang, Biao Yan, Zhenhua Wang

**Affiliations:** ^1^College of Information Science, Shanghai Ocean University, Shanghai, China; ^2^The Affiliated Eye Hospital, Nanjing Medical University, Nanjing, China; ^3^Eye Institute, Eye and ENT Hospital, Shanghai Medical College, Fudan University, Shanghai, China

**Keywords:** diabetic macular edema, optical coherence tomography, deep learning, DeepLabV3+, extraction model

## Abstract

**Introduction:**

Diabetic macular edema (DME) is a major cause of vision impairment in the patients with diabetes. Optical Coherence Tomography (OCT) is an important ophthalmic imaging method, which can enable early detection of DME. However, it is difficult to achieve high-efficiency and high-precision extraction of DME in OCT images because the sources of OCT images are diverse and the quality of OCT images is not stable. Thus, it is still required to design a model to improve the accuracy of DME extraction in OCT images.

**Methods:**

A lightweight model (DME-DeepLabV3+) was proposed for DME extraction using a DeepLabV3+ architecture. In this model, MobileNetV2 model was used as the backbone for extracting low-level features of DME. The improved ASPP with sawtooth wave-like dilation rate was used for extracting high-level features of DME. Then, the decoder was used to fuse and refine low-level and high-level features of DME. Finally, 1711 OCT images were collected from the Kermany dataset and the Affiliated Eye Hospital. 1369, 171, and 171 OCT images were randomly selected for training, validation, and testing, respectively.

**Conclusion:**

In ablation experiment, the proposed DME-DeepLabV3+ model was compared against DeepLabV3+ model with different setting to evaluate the effects of MobileNetV2 and improved ASPP on DME extraction. DME-DeepLabV3+ had better extraction performance, especially in small-scale macular edema regions. The extraction results of DME-DeepLabV3+ were close to ground truth. In comparative experiment, the proposed DME-DeepLabV3+ model was compared against other models, including FCN, UNet, PSPNet, ICNet, and DANet, to evaluate DME extraction performance. DME-DeepLabV3+ model had better DME extraction performance than other models as shown by greater pixel accuracy (PA), mean pixel accuracy (MPA), precision (Pre), recall (Re), F1-score (F1), and mean Intersection over Union (MIoU), which were 98.71%, 95.23%, 91.19%, 91.12%, 91.15%, and 91.18%, respectively.

**Discussion:**

DME-DeepLabV3+ model is suitable for DME extraction in OCT images and can assist the ophthalmologists in the management of ocular diseases.

## 1. Introduction

Diabetic macular edema (DME) is the major cause of vision loss in the patients with diabetic retinopathy. Increasing prevalence of DME is tightly correlated with the global epidemic of diabetes mellitus (1, 2). DME is usually caused by the rupture of retinal barrier and increased permeability of retinal vessels, which is characterized by the leakage of fluid and other plasma components. The effusion can accumulate in the macula, resulting in edema (3, 4). In the clinical work, the presence and severity of retinopathy are required to be determined according to the size of edema area.

**Figure 1:**
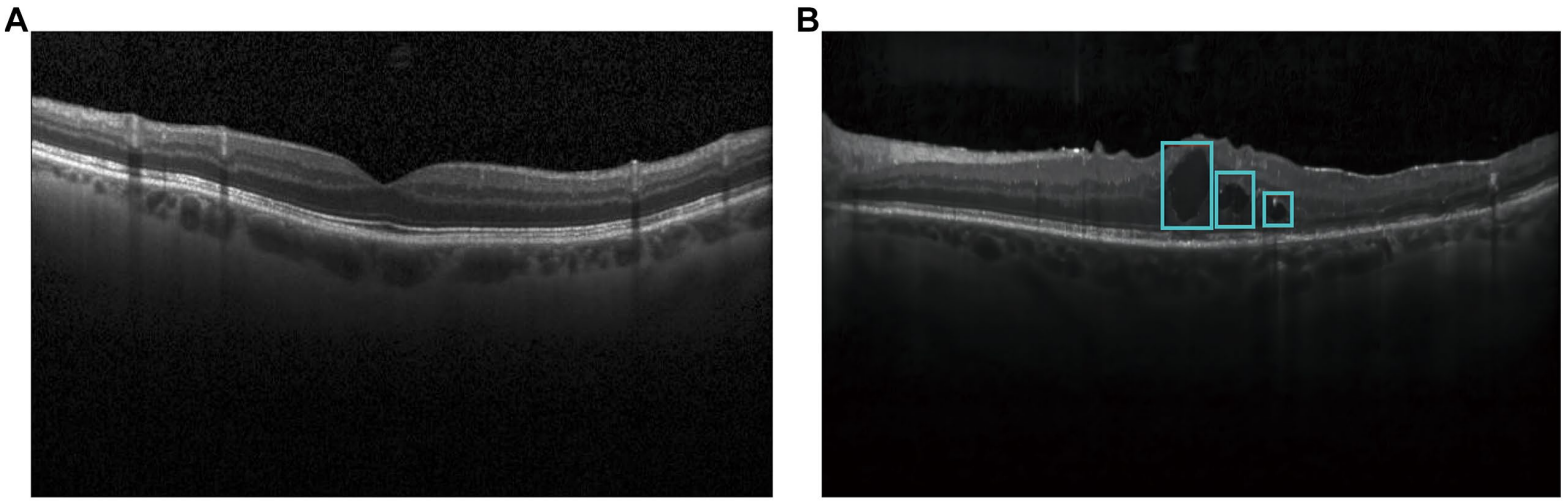
Optical coherence tomography images in diabetic patients and healthy controls. **(A)** Normal OCT image; **(B)** OCT image with DME.

Optical Coherence Tomography (OCT) is a non-contact, non-invasive, and highly sensitive ophthalmic imaging method, which can enable early detection of diabetic macular edema by observing the transverse section of macular degeneration (5). Normal OCT image is shown in Figure 1A and OCT image with DME is shown in Figure 1B. DMEs accumulated in typical relative positions within the main retinal layers. Based on OCT patterns of DME, DME can be classified into three different patterns, including diffuse retinal thickening (DRT), cystoid macular edema (CME), and serous retinal detachment (SRD). CME normally starts to manifest symptoms in the inner retina, while SRD and DRT typically appear in the outer retina. In the severe advanced stages of DR, CMEs can also proliferate from the inner to the outer retina and merge with DRT (6). Thus, rapid and accurate detection of all types of edemas is of great significance for evaluating the progression of diabetic retinopathy. In the clinical work, DME is usually segmented by the well-trained experts (7). However, manual extraction of DME edemas is time-consuming and labor-intensive. Moreover, there is inevitable variability in the extraction results by different experts. With increased prevalence of diabetes, an increasing number of patients require disease management based on OCT images in the clinical practices. Thus, it is highly required to design an automatic method for rapid and accurate detection of DME in OCT images.

Image extraction is processed and analyzed according to the features, including image color, spatial structure, and texture information (8). Image extraction models can divide an image into several specific regions, such as threshold-based extraction model (9, 10), region-based extraction model (11, 12), and edge detection-based extraction model (13). With the development of deep learning, several models have been developed to extract DME, such as fully convolutional network (FCN), U-Net, and PSPNet. Based on these deep learning models, several scholars have also developed the improved models for DME extraction. Table 1 showed the strengths and weaknesses of different models for DME extraction.

**Table 1:** Strengths and weaknesses of different models for DME extraction.

Models	Strengths	Weaknesses
FCN (14)	End-to-end pixel-level classification without inputting size constraints	Ignore target boundary details and lack spatial consistency
U-Net (15)	Good extraction performance on small objects	Down-sampling operators cause spatial information loss during encoding
PSPNet (16)	Aggregate contextual information from different regions and improve the ability of obtaining global information	No effective fusion of shallow features and missing target boundary details
FCN + Sobel operator + Dijkstra algorithm (17)	Achieve better results in DICE index	Divide OCT extraction tasks into two stages, coarse and fine extraction, which makes OCT extraction cumbersome
FCN + multiphase level set (18)	Avoid overlapping phenomenon of boundary and reduce the need for large training datasets	
U-Net + Bayesian deep learning (19)	Improve the accuracy of OCT image extraction with better versatility and interpretability	Poor extraction performance for small-area objects
PSPNet + dual attention mechanism (20)	Aggregate context information of different regions	Insensitive to the information of fluid accumulation regions in DME

The sources of OCT images are diverse and the quality of OCT images is not always stable. Moreover, the size and distribution of DMEs are not uniform and the borders of DMEs are blurred. Thus, it is still required to design a novel model to improve the accuracy of DME extraction in OCT images. In this study, we proposed a lightweight automatic model (DME-DeepLabV3+) based on the DeepLabV3+ architecture. The major contributions of the proposed DME-DeepLabV3+ are shown below:

Taking MobileNetV2 as the backbone, the ability of DME-DeepLabV3+ is improved in extracting the low-level features of DME.
Improving ASPP by the sawtooth wave-like dilation rate, DME-DeepLabV3+ avoids grid effects, learns more local information, and extracts high-level features of DME better.
Based on the decoder, DME-DeepLabV3+ fuses the low-level and high-level features of DME, and refines the results of DME extraction.

## 2. Materials and methods

**Figure 2:**
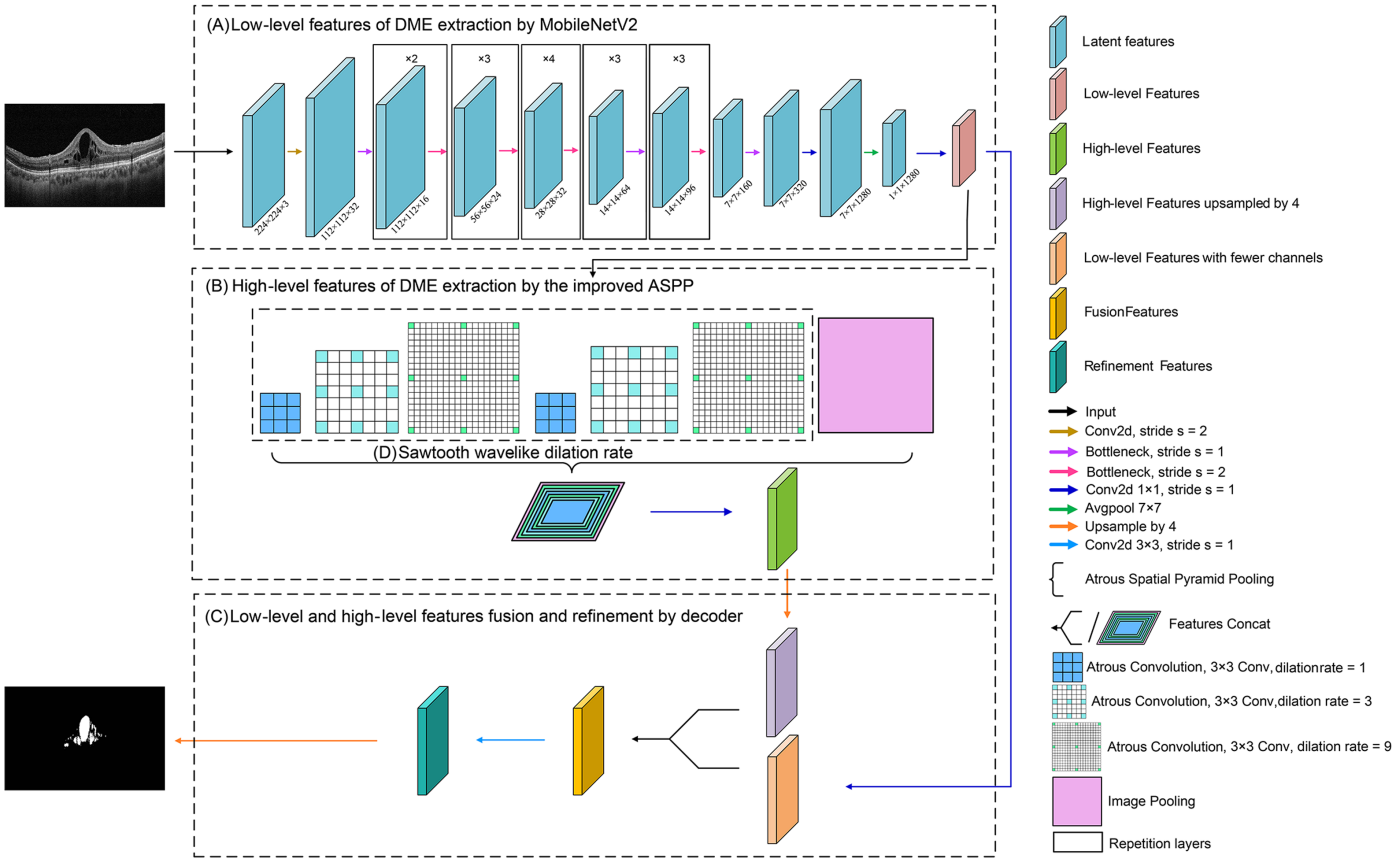
Flowchart of DME extraction by DME-DeepLabV3+ model.

The flowchart of the proposed model, DME-DeepLabV3+, was shown in Figure 2.

Low-level features of DME extraction by MobileNetV2; High-level features of DME extraction by the improved ASPP; Fusion and refinement of low-level and high-level features of DME by the decoder.

### 2.1. Low-level features of DME extraction by MobileNetV2

DeepLabV3+ is a deep learning model for image extraction with deep convolutional nets, which takes Xception as the backbone network (21). Xception uses numerous parameters, complicated operations, and high computer performance requirements (22), which leads to several challenges for DME extraction, such as fault-extraction and over-extraction problems. MobileNetV2 is a lightweight network, which shows a great advantage to solve the fault-extraction and over-extraction problems (23). In DME-DeepLabV3+ model, we used MobileNetV2 as the backbone to simplify model structure, which could improve the extraction efficiency and reduce the problems of fault-extraction and over-extraction.

MobileNetV2 used depthwise separable convolution to reduce the number of parameters and complex operations. Depthwise separable convolution consisted of DepthWise (DW) and PointWise (PW), whereas DW performed convolution operations on each channel of the input layer and PW fused the features and obtained the feature information with stronger expressive ability. MobileNetV2 used Inverted Residual to improve the memory efficiency.

**Figure 3:**
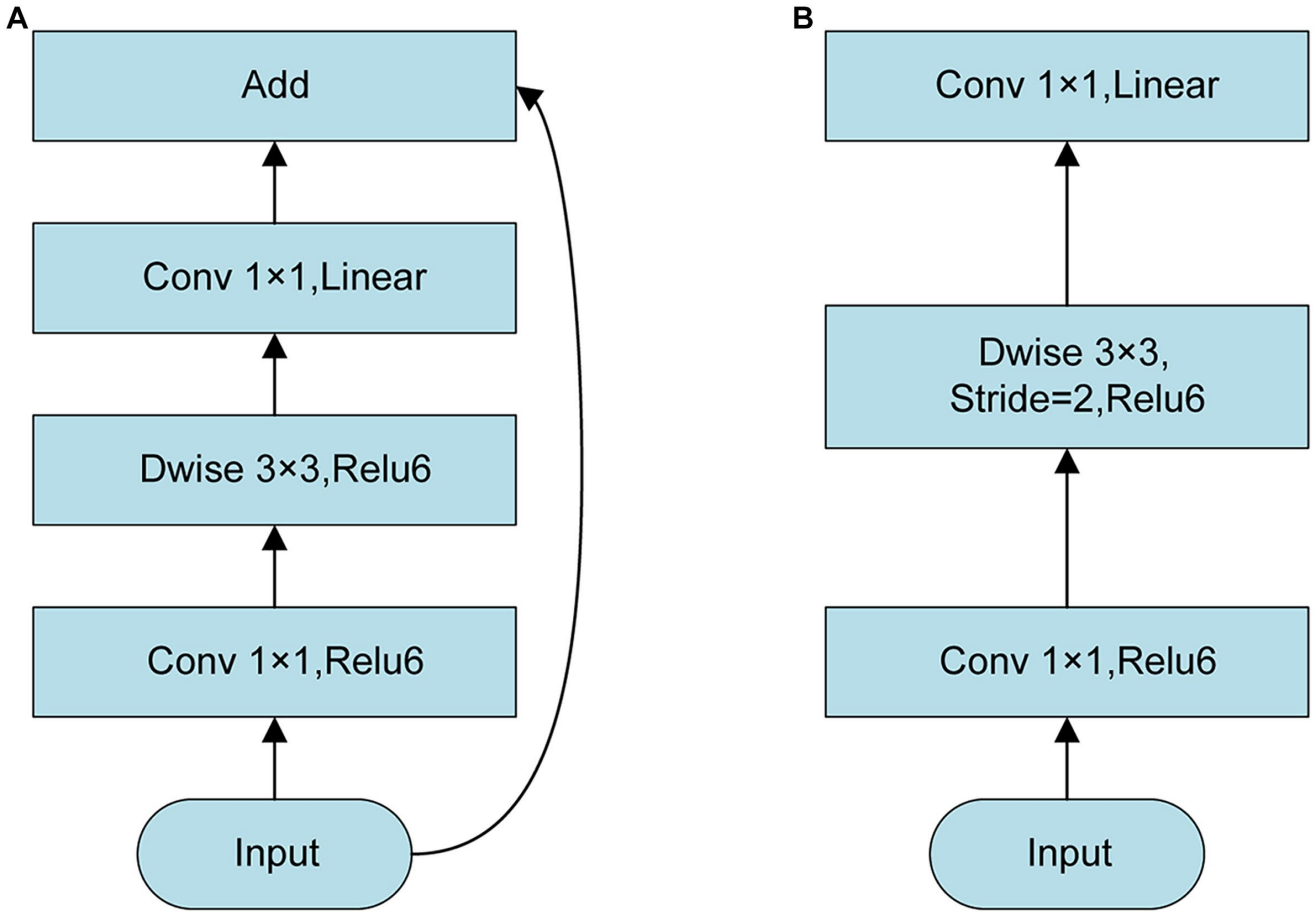
Structure of Inverted Residual. **(A)** Stride = 1; **(B)** Stride = 2.

In Inverted Residual, the dimension of DME features was increased by 1 × 1 convolution. Next, DME features were extracted by 3 × 3 DW convolution, and the dimension of DME features was reduced by 1 × 1 convolution (Figure 3). When the stride was 1, DME output features were consistent with the input features and shortcuts were used to add the elements of DME input and output. When the stride was 2, no shortcut was required. At the same time, a linear bottleneck neural network was used in the last 1 × 1 convolutional layer of Inverted Residual, which could reduce the loss of low-dimensional feature of DME information.

Compared with DeepLabV3+ with Xception as the backbone network, DME-DeepLabV3+ with MobileNetV2 as the backbone network not only improved the accuracy but also improved the efficiency in DME extraction.

### 2.2. High-level features of DME extraction by the improved ASPP

ASPP consists of atrous convolution with different dilation rates, which strikes the best trade-off between multi-scale feature extraction and context assimilation, especially for small objects (24). DME has multi-scale features, especially with several small areas of edema. ASPP was then used to extract high-level features of DME. However, the dilation rate in ASPP had a grid effect, which not only lost the semantic information but also ignored the consistency of local information in edema regions (25). Here, we replaced the original dilation rate with the sawtooth wave-like dilation rate to improve ASPP for extracting the high-level features of DME. A sawtooth wave-like dilation rate was formed by the repeated combination of two sets of the same “rising edge” type dilation rate.

**Figure 4:**
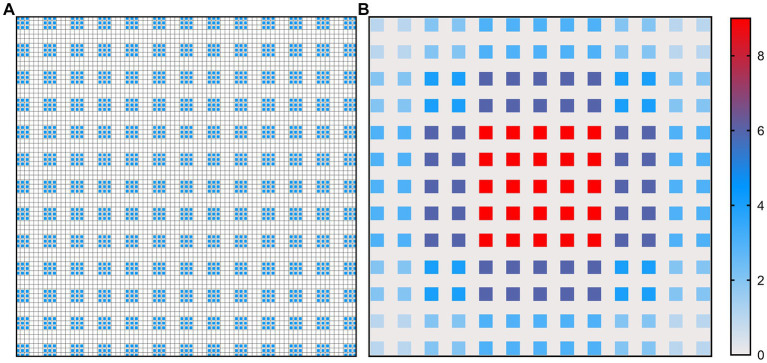
Illustration of atrous convolution principle of DeepLabV3 + with  dilation rate = [1, 6, 12, 18] and RF  =  75  ×  75. **(A)** Effective pixels in RF, which were marked in blue; **(B)** The number of calculation times of each pixel.

**Figure 5:**
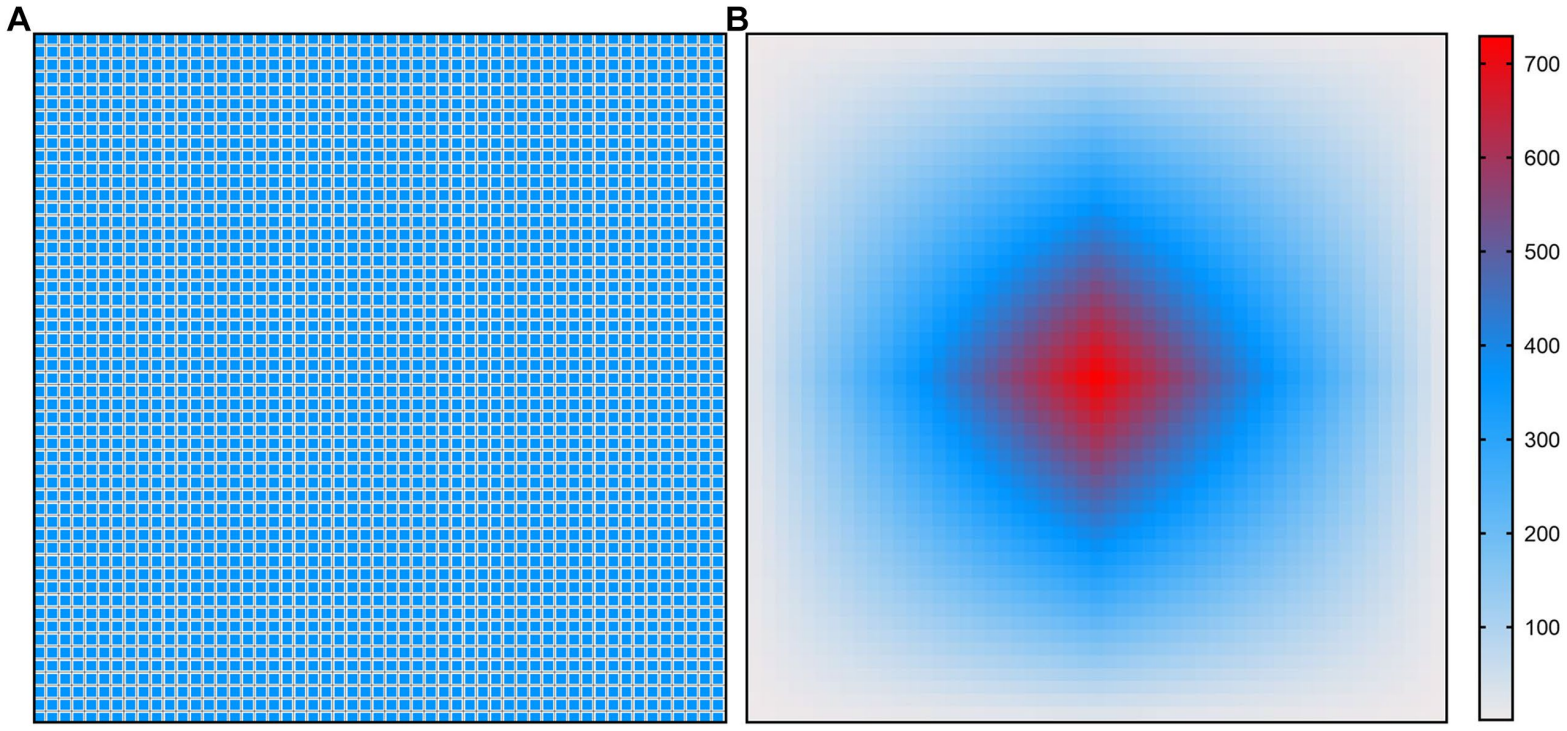
Illustration of atrous convolution principle of DME-DeepLabV3+ with dilation rate = [1, 3, 9, 1, 3, 9] and RF  =  53  ×  53. **(A)** Effective pixels in RF, which were marked in blue; **(B)** The number of calculation times of each pixel.

Figures 4, 5 show the illustration of the atrous convolution principle of DeepLabV3+ and DME-DeepLabV3+, respectively. Figures 4A,B show RF (receptive field) and the number of calculation times of DeepLabV3+. Figures 5A,B show RF and the number of calculation times of DME-DeepLabV3+. The results show that there was about 73% of information loss due to the grid effect in DeepLabV3+ model. In DME-DeepLabV3+ model, each pixel was effectively used and involved in further computations. Compared with DeepLabV3+ model, increased dilation rate in DME-DeepLabV3+ model can avoid the grid effects and learn more local information.

### 2.3. Fusion and refinement of low-level and high-level features by the decoder

Low-level and high-level features of DME were extracted by MobileNetV2 and the improved ASPP, respectively. All features of DME were fused and refined by the decoder. The decoder is mainly composed of ordinary convolution and fusion layers. It fuses the features extracted from the encoder, uses the up-sampling to restore the feature dimension, and outputs the prediction results of the same size with less information loss as possible (26). In the decoder, low-level features with fewer channels were obtained by 1 × 1 convolution. Bilinear up-sampling of high-level features were conducted by a factor of 4. The concatenation features were obtained by concatenating the low-level features and high-level features and a feature concatenation was refined by a few 3 × 3 convolutions. Finally, the results of DME extraction were output following another bilinear up-sampling by a factor of 4.

### 2.4. Ethical statement

The design and conduct of this study adhere to the intent and principles of the Declaration of Helsinki. The protocols were also reviewed and approved by the ethical committee of Eye Hospital (Nanjing medical university). Informed consents were obtained from all participants.

### 2.5. Datasets

**Figure 6:**
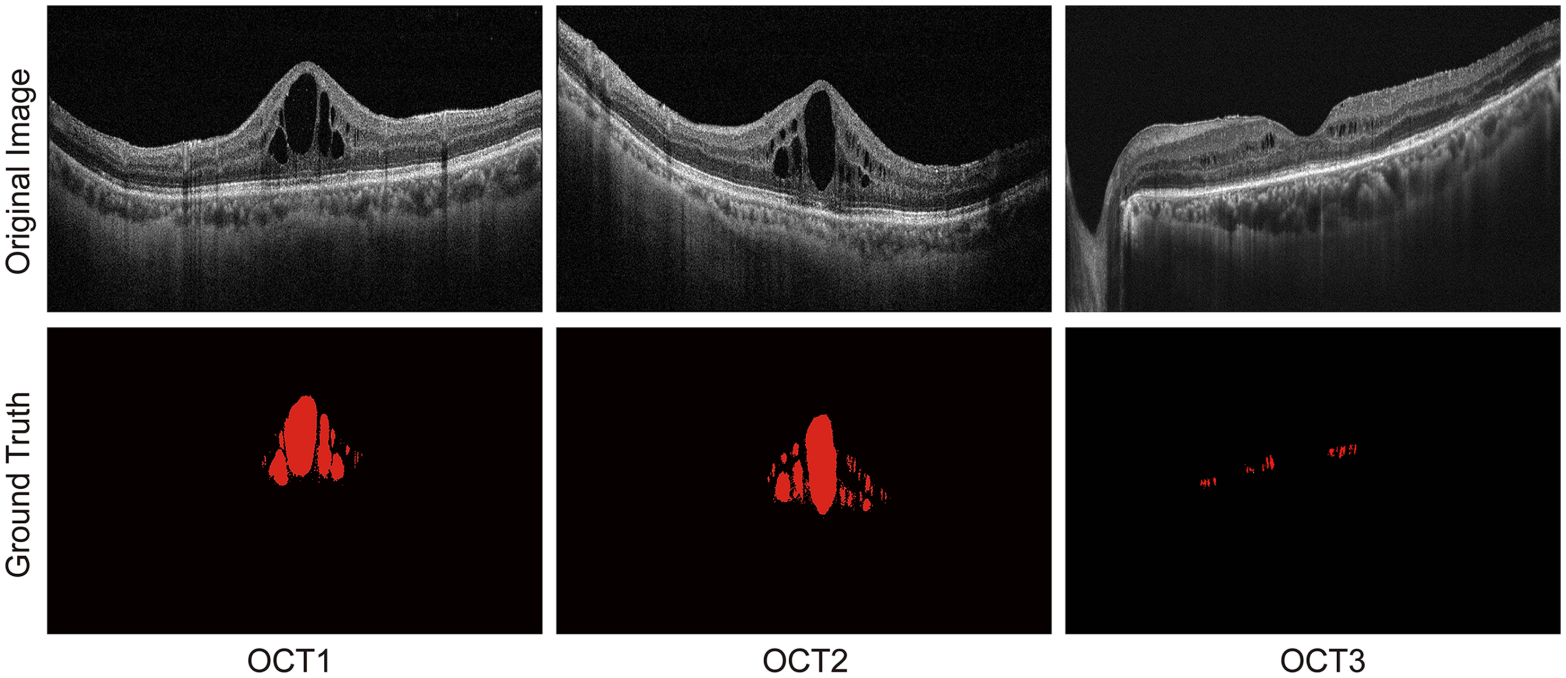
Original OCT images and DME labeling by three experienced clinicians.

The datasets contained 1711 OCT images, including 416 images (512 × 512 pixels) selected from the Kermany dataset (27) and 1,295 OCT datasets (938 × 420 pixels) collected from the Affiliated Eye Hospital, Nanjing Medical University. All patients were required to undergo OCT scanning by a spectral domain OCT (RTVue, Optovue Inc., United States). These OCT images were centered on the macula with an axial resolution of 10 μm and a 24-bit depth and were acquired in 2 s, covering 4 × 4 mm area. Inclusion criteria were as follows: the presence of macular edema in at least one eye and clear optical media allowing OCT imaging with good quality. Subsets of 1,369, 171, and 171 OCT images were randomly selected for training, validation, and testing, respectively. Each OCT image was individually labeled by three experienced clinicians who had more than 10-year clinical working experience. The annotation results were binarized by MATLAB software, where the background was labeled as 0 and the DME labeled as 1. Due to the limited human energy, some artificial deviations were inevitable. For these images, a senior expert was consulted and thorough rounds of discussion and adjudication were conducted to ensure the accuracy of the labeling. The original OCT images and ground truth are shown in Figure 6.

## 3. Implementation

The hardware configurations used for this study are shown below: Windows 10, NVIDIA GeForce RTX 3060. The software environment is the deep-learning framework PyTorch 1.10.0, CUDA 11.3, and the programming language Python 3.9.

## 4. Evaluation metrics

Seven metrics were calculated to estimate the extraction performance of DME-DeepLabV3+, including pixel accuracy (PA), mean pixel accuracy (MPA), precision (Pre), recall (Re), F1-score (F1), mean intersection over union (MIoU), and frames per second (FPS).


(1) $$\mathrm{PA}=\frac{\sum_{i=0}^{k}p_{\mathrm{ii}}}{\sum_{i=0}^{k}\sum_{j=0}^{k}p_{\mathrm{ij}}}$$

(2) $$\mathrm{MPA}=\frac{1}{k+1}\overset{k}{\underset{i=0}{\sum }}\frac{p_{\mathrm{ii}}}{\sum_{j=0}^{k}p_{\mathrm{ij}}}$$

(3) $$\mathrm{Pre}=\frac{\mathrm{TP}}{\mathrm{TP}+\mathrm{FP}}$$

(4) Re=TPTP+FN

(5) F1=2×Pre×RePre+Re

(6) $$\mathrm{MIoU}=\frac{1}{k+1}\overset{k}{\underset{i=0}{\sum }}\frac{p_{\mathrm{ii}}}{\sum_{j=0}^{k}p_{\mathrm{ij}}+\sum_{j=0}^{k}p_{\mathrm{ji}}-p_{\mathrm{ii}}}$$

(7) $$\mathrm{FPS}=\frac{\mathrm{frameNum}}{\mathrm{elapsedTime}}$$

TP, FP, and FN denote the true positive region, false positive region, and false negative region, respectively. $p_{ii}$ is the number of edema area pixels which was correctly classified as edema areas; $p_{ij}$ is the number of background area pixels which are misclassified as edema areas; $p_{ji}$ is the number of edema area pixels which are incorrectly classified as the background; k is the labeling results of different classes, where k = 0 expressed as background class and k = 1 as DME class; frameNum is the number of OCT images that are input to the model when performing inference; elapsedTime is the time consumed by the model when performing inference. PA is the overall pixel accuracy. MPA is the average pixel accuracy of DME and background. Pre and Re are the proportion of real DME regions in the samples predicted as DME and the proportion of correct predictions in all DME, respectively. F1-score (F1) is a balanced metric and determined by precision and recall. MIoU is a metric to measure the similarity of ground truth and prediction. FPS is the number of OCT images inferred per second.

## 5. Results

To evaluate the performance of DME extraction of DME-DeepLabV3+ model, two comparative experiments were performed. In experiment 1, DME extraction performance of DME-DeepLabV3+ model was evaluated by comparing against DeepLabV3+ model under different settings. In experiment 2, DME extraction performance of DME-DeepLabV3+ model was evaluated by comparing against other end-to-end models, including FCN, UNet, PSPNet, ICNet, and DANet.

### 5.1. Experiment 1 (ablation experiment)

**Figure 7:**
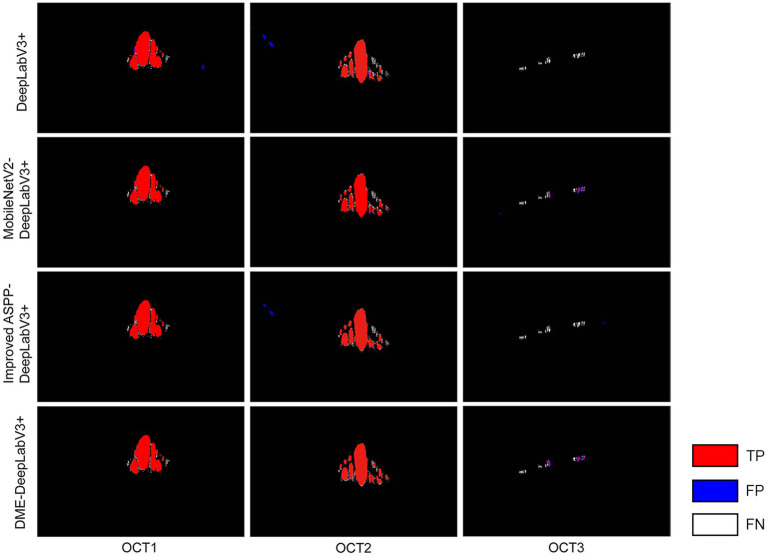
DME extraction results by DeepLabV3+ model with different settings Red, blue, and white DME regions represented TP regions, FP regions and FN regions.

To evaluate the effects of MobileNetV2 and the improved ASPP on DME extraction performance, the proposed DME-DeepLabV3+ model was compared against DeepLabV3+ model with different settings, including DeepLabV3+, DeepLabV3+ with MobileNetV2 (MobileNetV2-DeepLabV3+), DeepLabV3+ with the improved ASPP (Improved ASPP-DeepLabV3+). Figure 7 showed the DME extraction results by DeepLabV3+ model with different settings, where red, blue, and white DME regions represented true positive (TP) regions, false positive (FP) regions and false negative (FN) regions, respectively. DeepLabV3+ model led to some missed and false extraction of DME. MobileNetV2-DeepLabV3+ and improved ASPP-DeepLabV3+ reduced the missed and false extraction of DME. However, the missed extraction still existed in small edematous regions as shown in OCT2. DME-DeepLabV3+ had better extraction performance, especially in small-scale macular edema regions. The extraction results of DME-DeepLabV3+ were close to the ground truth.

Table 2 showed the results of evaluation metrics for DeepLabV3+ under different settings. Compared with DeepLabV3+ model, the MobileNetV2-DeepLabV3+ enhanced the scores of PA, MPA, Pre, Re, F1, MIoU, and FPS of DME extraction results, which were 98.69(0.28↑), 94.95(1.50↑), 91.02(0.57↑), 91.09(3.31↑), 91.06(1.97↑), 91.05(1.78↑), and 9.24(3.40↑), respectively. FPS increased by about 58%. The improved ASPP-DeepLabV3+ enhanced the scores of PA, MPA, Re, F1, and MIoU of DME extraction results, which were 98.47(0.06↑), 93.90(0.45↑), 88.49(0.71↑), 89.45(0.36↑), and 89.61(0.34↑), respectively. DME-DeepLabV3+ enhanced the scores of PA, MPA, Pre, Re, F1, and MIoU of DME extraction results, which were 98.71(0.30↑), 95.23(1.78↑), 91.19(0.74↑), 91.12(3.34↑), 91.15(2.06↑), and 91.18(1.91↑), respectively. FPS was 9.03, which was lower than that of MobileNetV2-DeepLabV3+ (0.21↓).

**Table 2:** Evaluation metrics of DME extraction by DeepLabV3+ model with different settings.

Models	Evaluation metrics						
	PA(%)	MPA(%)	Pre(%)	Re(%)	F1(%)	MIoU(%)	FPS(it/s)
DeepLabV3+	98.41 ± 0.07	93.45 ± 0.36	90.45 ± 0.85	87.78 ± 0.62	89.09 ± 0.16	89.27 ± 0.20	5.84 ± 0.87
MobileNetV2-DeepLabV3+	98.69 ± 0.01	94.95 ± 0.14	91.02 ± 0.58	91.09 ± 0.43	91.06 ± 0.08	91.05 ± 0.05	9.24 ± 0.33
Improved ASPP-DeepLabV3+	98.47 ± 0.03	93.90 ± 0.40	90.43 ± 0.30	88.49 ± 0.68	89.45 ± 0.42	89.61 ± 0.25	5.59 ± 0.13
DME-DeepLabV3+	98.71 ± 0.02	95.23 ± 0.26	91.19 ± 0.44	91.12 ± 0.48	91.15 ± 0.02	91.18 ± 0.09	9.03 ± 0.43

### 5.2. Experiment 2 (comparative experiment)

**Figure 8:**
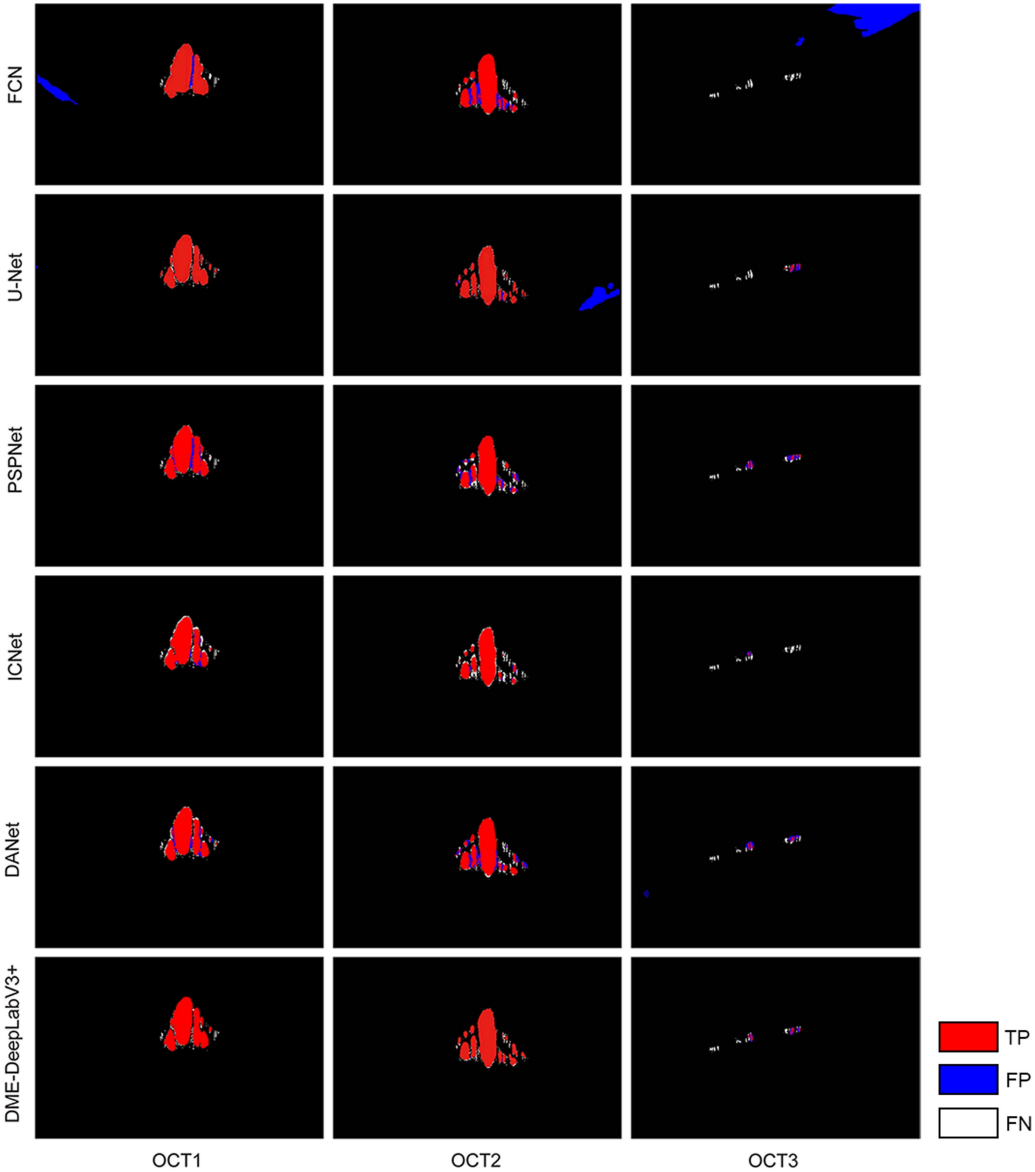
DME extraction results by different models Red, blue, and white DME regions represented TP regions, FP regions and FN regions.

We evaluate DME extraction performance of DME-DeepLabV3+ model by comparing against other models, including FCN, UNet, PSPNet, ICNet, and DANet. Figure 8 showed DME extraction results by different models, where red, blue, and white DME regions represented true positive (TP) regions, false positive (FP) regions, and false negative (FN) regions, respectively. As shown in Figure 8, DME extraction results of DME-DeepLabV3+ were close to the ground truth. A part of the background was extracted falsely by FCN, U-Net, or DANet models. Compared with FCN and U-Net, PSPNet and ICNet reduced the fault-extraction, but the small-scale macular edema was over-extracted. Table 3 showed the parameter configurations of different models and Table 4 showed the results of DME evaluation metrics. Compared with FCN, U-Net, PSPNet, and DANet models, DME-DeepLabV3+ achieved higher scores of PA, MPA, and FPS. As for Pre, Re, F1, and MIoU, DME-DeepLabV3+ substantially exceeded other models. Compared with ICNet, DME-DeepLabV3+ achieved a better trade-off in the accuracy and efficiency for DME extraction.

**Table 3:** Parameter configurations of different DME extraction models.

Models	Backbone	Learning rate	Total epochs	Batch size
FCN	ResNet50	0.01	200	1
U-Net	VGG16	0.01	200	2
PSPNet	ResNet50	0.01	200	2
ICNet	ResNet50	0.01	200	2
DANet	ResNet101	0.0001	200	2
DME-DeepLabV3+	MobileNetV2	0.01	200	2

**Table 4:** Evaluation metrics of DME extraction by different models.

Models	Evaluation metrics						
	PA(%)	MPA(%)	Pre(%)	Re(%)	F1(%)	MIoU(%)	FPS(it/s)
FCN	98.27 ± 0.11	89.88 ± 1.50	81.76 ± 1.20	79.44 ± 1.40	80.58 ± 0.85	82.66 ± 0.48	4.08 ± 0.20
U-Net	98.61 ± 0.01	90.73 ± 1.05	86.31 ± 0.41	81.15 ± 0.65	83.58 ± 0.15	85.32 ± 0.23	3.43 ± 0.24
PSPNet	98.69 ± 0.04	92.52 ± 0.41	84.61 ± 1.27	85.76 ± 0.90	85.17 ± 0.24	86.41 ± 0.21	7.96 ± 0.48
ICNet	98.07 ± 0.02	90.94 ± 0.20	90.73 ± 0.78	82.57 ± 0.93	86.45 ± 0.29	86.86 ± 0.13	15.86 ± 0.30
DANet	98.06 ± 0.01	92.15 ± 0.16	87.44 ± 0.77	85.68 ± 1.16	86.54 ± 0.21	87.11 ± 0.08	6.12 ± 0.50
DME-DeepLabV3+	98.71 ± 0.02	95.23 ± 0.26	91.19 ± 0.44	91.12 ± 0.48	91.15 ± 0.02	91.18 ± 0.09	9.03 ± 0.43

## 6. Conclusion and discussion

With the increased incidence of diabetes, DME has become a major cause of visual impairment in diabetic patients (28). DME occurs as a result of the disruption of blood-retinal barrier and consequent increase in vascular permeability (29). OCT allows longitudinal, functional, and microstructural analysis of human macula (30). However, manual labeling DME is time-consuming and labor-intensive (31). Automatic extraction of DME based on machine learning can help physicians assess disease severity, determine treatment options, and improve life quality of patients (32). Thus, it is urgent to develop an efficient model for DME detection. In this study, we proposed a lightweight model based on DeepLabV3+, termed DME-DeepLabV3+, to extract DME in OCT images. MobileNetV2 architecture was used as the backbone to extract the low-level features of DME and reduce the model complexity to enhance DME detection accuracy. With the help of improved ASPP structure, DME-DeepLabV3+ avoided the grid effects and learned more local information. Finally, the decoder was used to fuse the low-level and high-level features of DME and refined the results of DME extraction.

OCT image modality has been widely used for detecting DME due to its non-invasive and high-resolution features. Considering the clinical characteristics that are present in OCT images such as thickness, reflectivity or intraretinal fluid accumulation, DMEs have been categorized into three different types: SRD, DRT, and CME. Traditional DME detection is based on the low-level hand-crafted features, which require significant domain knowledge and are sensitive to the variations of lesions. Given great variability of morphology, shape, and relative ME position, it is difficult to detect all three ME types simultaneously. Our proposed model can achieve automatic and simultaneous detection of all three types of ME (SRD, DRT, and CME) in the ophthalmological field. However, the accuracy of DRT detection is still not good as SRD or CME detection. DRT is characterized by a sponge-like retinal swelling of the macula with reduced intraretinal reflectivity. In addition, DRT is characterized by uniform thickening of inner retinal layers but without macroscopic optical empty spaces. Thus, further improvement of our proposed model is still required for enhancing the accuracy of the automatic detection of DRT edemas.

In clinical practice, layer segmentation and fluid area segmentation can provide qualitative information and visualization of retinal structure, which is important for DME assessment and monitoring. Although commercial OCT devices with on-board proprietary segmentation software are available, the definition of retinal boundaries varies between the manufacturers, making the quantitative retinal thickness difficult. In addition, proprietary software is difficult to be used for image analysis from other OCT devices, which poses a great challenge for effective diagnosis of DME (33). Although automated methods for layer segmentation have been proposed, most of them usually ignore the priority of mutually exclusive relationships between different layers, which can also affect the accuracy of DME assessment (34). In future study, we will improve our model to consider both layer segmentation and fluid area segmentation for better monitoring the progression of DME in retinal diseases.

Both microaneurysm (MA) formation and DME lesions are the important signs of DR. Early and accurate detection of DME and MAs can reduce the risk of DR. Due to the small size of MA lesions and low contrast between MA lesion and retinal background, automated MA detection is still challenging. Many imaging modalities have been used to detect MAs, including color fundus images, optical coherence tomography angiography (OCTA), and fluorescein fundus angiography (FFA). However, MAs are situated on the capillaries, which are not often visible in color fundus images. Although FFA can capture the small changes of retinal vessels, FFA is an invasive method compared with other imaging modalities. OCTA can provide the detailed visualization of vascular perfusion and allow for the examination of retinal vasculature in 3D (35). In future study, we would also improve our model by considering the segmentation of FFA for better monitoring the progression of DME in retinal diseases. We would design modules with better feature extraction capabilities, such as embedding attention mechanism to the model, strengthening key information, suppressing useless information, and better capturing contextual information, to improve the generalization of the model for the diagnosis of retinal diseases.

## Data availability statement

The raw data supporting the conclusions of this article will be made available by the authors, without undue reservation.

## Author contributions

BY, ZW, and QJ were responsible for the conceptualization and data collection. BY, ZW, and YB were responsible for the experiment design and manuscript writing. JL and LS conducted the data collection and data entry. BY and ZW were responsible for overall supervision and manuscript revision. All authors contributed to the article and approved the submitted version.

## Funding

This research was generously supported by the grants from the National Natural Science Foundation of China (Grant Nos. 82171074 and 82070983).

## Conflict of interest

The authors declare that the research was conducted in the absence of any commercial or financial relationships that could be construed as a potential conflict of interest.

The reviewer ZH declared a shared parent affiliation with the authors LS, QJ, and BY to the handling editor at the time of review.

## Publisher’s note

All claims expressed in this article are solely those of the authors and do not necessarily represent those of their affiliated organizations, or those of the publisher, the editors and the reviewers. Any product that may be evaluated in this article, or claim that may be made by its manufacturer, is not guaranteed or endorsed by the publisher.
